# Associations between academic achievement and internalizing disorders in Sweden 2006–2018: Moderation by sex, socio-economic status, and country of birth

**DOI:** 10.1186/s12887-025-06301-4

**Published:** 2025-10-28

**Authors:** Björn Högberg, Karina Nilsson, Solveig Petersen, Mattias Strandh

**Affiliations:** 1 Department of Social Work, Umeå University, Umeå, SE-901 87 Sweden; 2 Centre for Demographic and Ageing Research, Umeå University, Umeå, Sweden; 3 Department of Sociology, Umeå University, Umeå, Sweden; 4 Department of Epidemiology and Global Health, Umeå University, Umeå, Sweden

**Keywords:** School performance, Grade point average, Anxiety disorders, Mood disorders, Temporal trends, Inequality.

## Abstract

**Background:**

Recent findings indicate that the association between academic achievement and internalizing mental health problems or disorders has become stronger over time. It is not known if this change has been driven by specific subgroups of students. The aim of this paper was to investigate temporal changes between 2006 and 2018 in the association between academic achievement and internalizing disorders among Swedish school year 9 students (aged 16 years) across subgroups defined by students’ sex, socio-economic status, and country of birth.

**Methods:**

Register data on all students (*N* = 1,422,487) graduating from the last year of Swedish compulsory school (school year 9) between 2006 and 2018 were used in the analyses. Achievement was measured by students’ final grade point average, and internalizing disorders were measured using data on treatment for anxiety or mood disorders. Logistic regression models were used to test for multiplicative interaction, and marginal effects for additive interaction, between achievement and time. Models were fitted separately for subgroups defined by sex, socio-economic status, and country of birth.

**Results:**

The association between achievement and treatment for internalizing disorders became stronger over time among Swedish-born students but was largely stable (in absolute terms) or diminished (in relative terms) among immigrant students. Among Swedish-born students, the largest increase in treatment for internalizing disorders was observed in low-achieving girls. Differences in trends depending on socio-economic status were more mixed.

**Conclusions:**

Low-achieving students and girls face multiple disadvantages in life. The disproportionate increase in internalizing disorders among low-achieving students, and especially low-achieving girls, is concerning from the perspective of equity in health.

**Supplementary Information:**

The online version contains supplementary material available at 10.1186/s12887-025-06301-4.

## Introduction

Academic achievement and internalizing mental health problems or disorders such as mood or anxiety disorders are strongly and bidirectionally related. Internalizing mental health problems or disorders can lead to lower academic achievement by undermining sleep, focus, concentration, and motivation, and by increasing the risk of absenteeism [1–8]. Low academic achievements, in turn, can increase the risk of internalizing problems or disorders by generating stress, worries, and fear, undermining self-worth, and by bringing about feelings of shame and stigma [9–15]. Considering these associations, it is important to understand if the increasing rates of internalizing problems or disorders reported among adolescents across high-income countries [16] are related to their academic achievement.

Recent findings from Sweden indicate that the association between achievement and treatment for internalizing or other psychiatric disorders has become stronger over time, such that the overall increase in such disorders has been disproportionately large among low-achieving students [17, 18]. It is not known whether this trend has been driven by specific subgroups of students. However, the association between achievement and internalizing disorders may differ depending on characteristics such as sex, socio-economic status, or country of birth. Since girls tend to value school higher [19] and be more dependent on their achievements in school for their labour market prospects [20], there are reasons to expect that academic failures has a disproportionate influence on their mental health. Existing research on differences between girls and boys in the association between achievement and internalizing problems has, however, reached discrepant results. Most studies have found no or small differences depending on sex [5, 21–23], other stronger associations for girls [4] or for boys [14, 24]. Moderation by social background or country of birth has been less intensely studied. As for social background, families with high socio-economic status may be better able to support children that struggle in school or with their mental health [25, 26], thereby weakening the association between achievement and internalizing problems. On the other hand, academic failure may be more stigmatizing and disruptive for students with high socio-economic status [26–28]. Existing research on socio-economic differences has reached mixed results: some studies have found that higher socio-economic status attenuates the association between academic achievement and internalizing problems [3], while others have found no or complex non-linear moderating effects [24, 29]. To the best of our knowledge, only one study has investigated moderation by country of birth: Hynek et al. [30] found that the association between academic achievement and mental healthcare service use was stronger among native (Norwegian) students compared to students with immigrant and non-western background.

Given this background, the aim of this paper was to investigate temporal changes between 2006 and 2018 in the association between academic achievement and treatment for internalizing disorders among Swedish school year 9 students (aged 16 years) across subgroups defined by student’s sex, socio-economic status, and country of birth.

## Materials and methods

### Data

Data from five Swedish administrative registers were used in the analysis. The National Patient Register contains data on specialized psychiatric inpatient and outpatient care in Swedish hospitals. The Prescribed Drug Register contains data on all prescribed drugs dispensed at Swedish pharmacies. The National Pupil Register contains data on all students enrolled in Swedish compulsory school, including their school year and grades. The Total Population Register contains data on sex, country of birth, and birth year of all Swedish residents. The Longitudinal Integrated Database for Health Insurance and Labour Market Studies (LISA) contains data on the highest attained education of the students’ parents. The data were made available through the Umeå SIMSAM Lab infrastructure [31], and approval of the use of the data for research purposes was granted by the Swedish Ethical Review Authority (Dnr 2023–03999-01 and Dnr 2023–05360-02).

### Population

All students graduating from the last year of Swedish compulsory school (school year 9) between 2006 and 2018 were included in the analyses. 2006 was chosen as starting year since this was the first year with complete data on pharmacological treatment. The sample size was 1,422, 487, although this was reduced in some models due to missing data on parental education. Grade retention and advancement is rare in Swedish schools, and 99.8% of the sample was aged 15–17 years. In cases where students graduated more than once from compulsory school (0.29% of the sample), only the first graduation year was included in the analysis.

### Academic achievement

Achievement was measured by students’ final grade point average (GPA) in year 9 of compulsory school. The 16, in some cases 17, best subject grades are summed at the end of year 9. The sum of grades functions as a grade point average, which we will subsequently denote as the students’ GPA. If an upper secondary school or program has more applicants than slots, students are, with a few exceptions, granted access based on their GPA. Moreover, eligibility to national programs in upper secondary school requires passing grades in certain subjects. Thus, final grades in year 9 carry high stake for Swedish students.

In this study, GPA was transformed into quintiles within each graduation year to account for changes in grading systems and grade inflation. Quintiles were chosen to balance the goal of allowing for a flexible functional form with the goal of keeping the number of interaction terms at a manageable level. Additional file A shows that the association between achievement and treatment for internalizing disorders is strongly curvilinear, with the by far highest rates of treated students observed in the bottom 20% of the GPA distribution and with more similar rates in the middle and upper parts of the distribution. In some analyses, the focus was therefore on the contrast between the 1 st quintile (low GPA) and the 2nd – 5th quintiles collapsed into one category (medium/high achievement).

### Internalizing disorders

Internalizing disorders were measured using data on treatment in specialized psychiatric inpatient or outpatient care as well as data on pharmacological treatment. Only the patient’s main diagnosis was used. There may be some underreporting of data from clinics to the National Patient Register [32]. Many of these cases may be captured by data on drug prescriptions from the Prescribed Drug Register, which has very high coverage and reliability [33]. Neither register includes patients who were only given drugs in clinics or patients that received non-pharmacological treatment in primary care.

The following codes were used to identify internalizing disorders: International Statistical Classification of Diseases 10 (ICD-10) codes F30-39 (mood disorders) and F40-44 (neurotic and stress-related disorders, including anxiety disorders), and Anatomical Therapeutic Chemical (ATC) classification system codes N05B (anxiolytics), N05C (hypnotics and sedatives) and N06A (antidepressants). Since some antidepressants are used to treat both anxiety and mood disorders, it was not possible to differentiate between these two disorders in the data. Students were coded 1 if they received a diagnosis or treatment according to the above definition at least once in the year that they graduated from compulsory school, and 0 otherwise.

### Moderators

The focal moderating variable was time and was measured by the year of graduation from compulsory school (2006–2018). Additional moderating variables were sex, country of birth, and socio-economic status. Sex was measured by legal sex (boy = 0; girl = 1). Country of birth was measured as the country of birth of the student (0 = born in Sweden, henceforth Swedish-born; 1 = not born in Sweden, henceforth immigrant). Socio-economic status was measured as the highest attained education by any parent at the time of graduation (0 = lower or upper secondary education; 1 = post-secondary education). Data on parental education was not available in 2018 since the LISA database did not extend beyond 2017 when data were delivered to the Umeå SIMSAM Lab.

### Statistical analysis

Logistic regression models were fitted with treatment for internalizing disorders as the outcome and GPA, year, as well as the interaction between GPA and year, as independent variables. The models were first fitted separately for Swedish-born and immigrant students. Separate models were then fitted for subgroups defined by combinations of both country of birth AND sex or socio-economic status (i.e., Swedish-born girls, Swedish-born boys, etc.). Multiplicative interaction [34] was assessed based on the product of the interaction between GPA and year, measured on the odds ratio scale. Multiplicative interaction means that the combined association between two exposures exceeds the *product* of their separate associations. Since low GPA was used as reference category, product terms larger than 1 indicate a stronger association between low achievement and treatment for internalizing disorders over time. Multiplicate interactions show *relative* changes over time. Additive interaction was assessed by transforming the odds into marginal effects of time (year) across GPA quintiles. The marginal effects thus indicate the average yearly change in the probability of treatment for internalizing disorders over time. Additive interaction means that the combined association of two exposures exceeds the *sum* of their separate effects. Thus, additive interactions show absolute changes over time.

### Supplementary analyses

Several additional analyses were also conducted. First, logistic regression models with three-way interactions between GPA, year and, respectively, sex, country of birth and socio-economic status were fitted to formally test the significance of the subgroup differences. Second, pooled logistic models with all subgroups combined, and with sex, country of birth and socio-economic status as control variables, were fitted. Third, multinomial logistic regression models with the GPA quintiles as outcome and treatment for internalizing disorders as an independent variable were fitted to investigate the reversed association. Fourth, only data on inpatient care were used to investigate if the associations were similar for more severe forms of internalizing disorders. Fifth, more fine-grained measurements of country of origin and parental education were used to investigate heterogeneity within the cruder groups used in the main analysis.

The aim of the study was to investigate associations, not estimate causal effects, and no control variables were included in the analyses [35]. 0.05 was used as significance level in the study. All analyses were conducted using Stata v 14.

## Results

Table 1 shows that the proportion treated for an internalizing disorder increased strongly over time and was markedly higher among low-achieving students. It was also higher among girls and Swedish-born students, and marginally higher among students with low socio-economic status.

**Table 1:** Summary statistics: number observations per category

	No internalizing disorder	Internalizing disorder (% of total in parentheses)	Total
Year			
2006	125,008	2,636 (2.1%)	127,644
2007	123,952	3,030 (2.4%)	126,982
2008	121,850	3,276 (2.6%)	125,126
2009	116,954	3,326 (2.8%)	120,280
2010	111,781	3,622 (3.1%)	115,403
2011	103,281	3,910 (3.6%)	107,191
2012	96,113	4,110 (4.1%)	100,223
2013	91,049	4,352 (4.6%)	95,401
2014	91,682	5,081 (5.3%)	96,763
2015	90,774	5,451 (5.7%)	96,225
2016	93,392	6,590 (6.6%)	99,982
2017	95,633	7,409 (7.2%)	103,042
2018	99,716	8,509 (7.9%)	108,225
GPA quintile			
1st	266,799	28,941 (9.8%)	295,740
2nd	277,260	11,226 (3.9%)	288,486
3rd	276,543	8,740 (3.1%)	285,283
4th	274,682	6,771 (2.4%)	281,453
5th	265,901	5,624 (2.1%)	271,525
Country of birth			
Swedish	1,223,447	56,472 (4.4%)	1,279,919
Immigrant	137,666	4,826 (3.4%)	142,492
Missing	72	4 (5.3%)	76
Sex			
Boy	707,629	23,329 (3.2%)	730,958
Girl	653,537	37,973 (5.5%)	691,510
Missing	19	0 (-)	19
Socio-economic status			
Low	617,441	27,134 (4.2%)	644,575
High	634,192	24,878 (3.8%)	659,070
Missing	109,552	9,290 (7.8%)	

Most observations with missing data on socio-economic status (99,716 of 118,842) are due to missing data in 2018. Internalizing disorder = diagnosed in specialized psychiatric inpatient or outpatient care and/or prescribed psychotropic drugs for internalizing problems

**Fig. 1:**
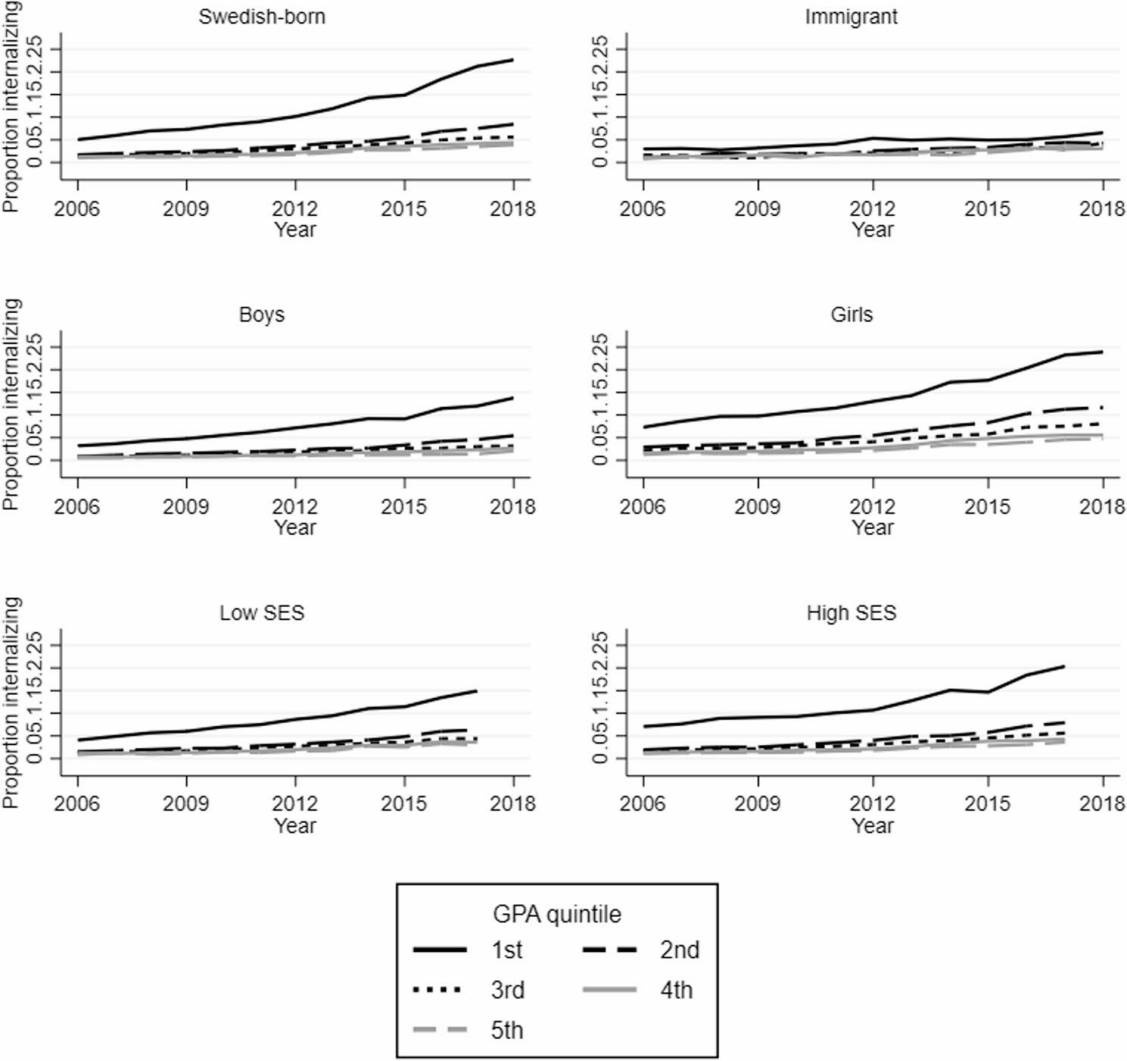
Proportion with internalizing disorder by year and GPA quintile. Abbreviations: GPA = grade point average; SES = socio-economic status. Internalizing disorder = diagnosed in specialized psychiatric inpatient or outpatient care and/or prescribed psychotropic drugs for internalizing problems

Figure 1 shows that the proportion treated for an internalizing disorder was, in all subgroups, highest in the lowest-achieving quintile already in 2006, but also that the increase in absolute terms was markedly greater in this quintile in all subgroups except for immigrants. The increase in absolute terms was also larger among girls than boys, and somewhat larger among students with high compared to low socio-economic status.

**Fig. 2:**
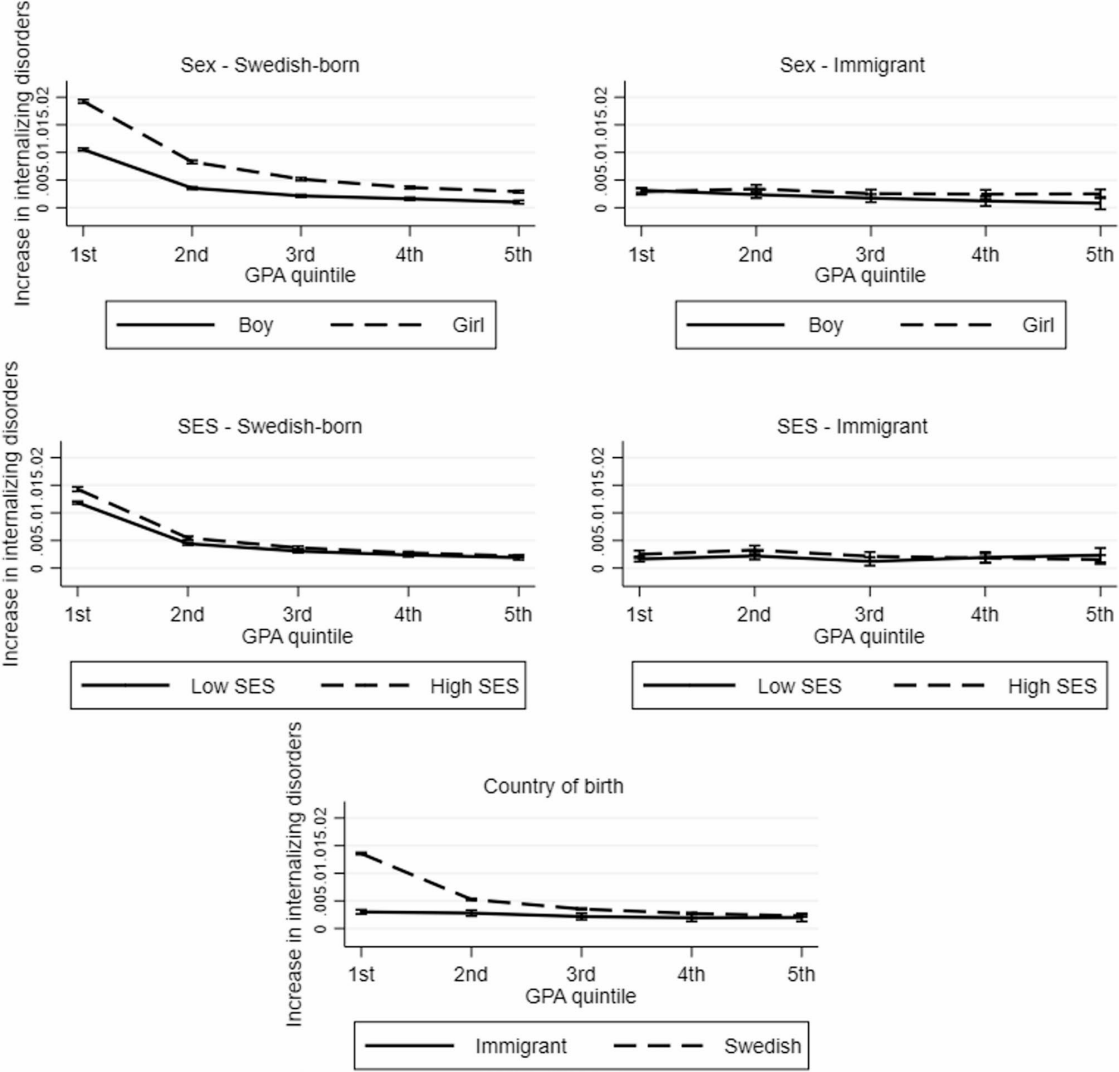
Marginal effect of year on probability of internalizing disorder, by GPA quintile. The y-axis shows the yearly increase in the probability of internalizing disorders. Abbreviations: GPA = grade point average; SES = socio-economic status. Internalizing disorder = diagnosed in specialized psychiatric inpatient or outpatient care and/or prescribed psychotropic drugs for internalizing problems

Figure 2 and Table 2 formally address the aim of the study. Fig. 2 shows average yearly changes in the probability being treated for an internalizing disorder (y-axis) across GPA quintiles (x-axis), thus investigating additive interaction. The slopes of the lines indicate the change across the full GPA distribution, while the difference in levels between the 1 st and the other GPA quintiles indicate if the increase was greater among low-achieving students. Tables with regression coefficients and formal significance tests are included in Additional file B. Starting with country of birth, the line for immigrant students is almost completely flat, while the line for Swedish-born students slopes downwards, especially between the 1^st^ and the 2^nd^ quintiles. This indicates that the association between achievement and treatment for internalizing disorders increased among Swedish-born but barely among immigrant students, and that this was primarily due to the disproportionate increase in the lowest quintile among Swedish-born students. The slope is also steeper among Swedish-born girls compared to Swedish-born boys, and, although both are relatively flat, marginally steeper among immigrant boys compared to immigrant girls. The slope is somewhat steeper for Swedish-born students with high compared to low socio-economic status, and basically flat for immigrant students regardless of socio-economic status. Additional file B shows that the increase, in absolute terms and thus in terms of additive interactions, in treatment for internalizing disorders was significantly greater in the lowest quintile compared to the 2nd −5th quintiles among all subgroups of Swedish-born students (pooled: *p* < 0.001; girls: *p* < 0.001; boys: *p* < 0.001; high socioeconomic status: *p* < 0.001; low socioeconomic status: *p* < 0.001) as well as among immigrants as whole (*p* = 0.011) and among immigrant boys (*p* < 0.001). Additional file C shows marginal effects from the corresponding three-way interactions. The increase in the gap between low- and medium/high achieving students was, in absolute terms, greater among Swedish-born compared to immigrant students (*p* < 0.001), among Swedish-born girls compared to boys (*p* < 0.001), among immigrant boys compared to girls (*p* = 0.012), and among Swedish-born students with high compared to low socio-economic status (*p* < 0.001).

**Table 2:** Odds of internalizing disorder by GPA and year. Stratified by country of birth, sex and SES

	Swedish-born				Immigrant			
	Girls	Boys	High SES	Low SES	Girls	Boys	High SES	Low SES
	OR [95% CI]	OR [95% CI]	OR [95% CI]	OR [95% CI]	OR [95% CI]	OR [95% CI]	OR [95% CI]	OR [95% CI]
Graduation year	1.135***	1.148***	1.136***	1.145***	1.108***	1.129***	1.101***	1.133***
	[1.131,1.139]	[1.142,1.154]	[1.131,1.141]	[1.139,1.151]	[1.093,1.124]	[1.107,1.151]	[1.082,1.120]	[1.108,1.159]
Low GPA	4.231***	4.480***	5.572***	3.479***	2.327***	3.465***	2.716***	3.283***
	[4.030,4.441]	[4.202,4.777]	[5.234,5.932]	[3.295,3.673]	[1.925,2.814]	[2.748,4.370]	[2.180,3.383]	[2.569,4.195]
Graduation year X Low GPA	1.023***	1.015***	1.002	1.016***	0.962***	0.963**	0.960**	0.932***
	[1.017,1.029]	[1.007,1.022]	[0.994,1.010]	[1.009,1.023]	[0.943,0.982]	[0.940,0.986]	[0.935,0.986]	[0.906,0.958]
Constant	0.016	0.006	0.012	0.011	0.013	0.006	0.013	0.007
N	624,194	655,725	605,082	581,711	67,292	75,200	53,969	62,848

Table presents results from logistic regression models. Internalizing disorder = diagnosed in specialized psychiatric inpatient or outpatient care and/or prescribed psychotropic drugs for internalizing problems

*Abbreviations*: *OR* Odds ratio, *CI* Confidence interval, GPA Grade point average, *SES* Socio-economic status

^*^p < 0.05

^**^p < 0.01

^***^p < 0.001

Table 2 shows the odds of treatment for internalizing disorders depending on year and GPA, thus investigating multiplicative interaction. For brevity, only comparisons between the 1 st (low GPA) and the 2nd −5th (medium/high GPA) is shown. Coefficients for all GPA quintiles are included in Additional file D. The multiplicative interaction is significant and positive for both Swedish-born girls (*p* < 0.001) and boys (*p* < 0.001), indicating a greater increase in treatment for internalizing disorders among low-achieving Swedish-born students in relative terms (i.e., relative to baseline differences in 2006) as well. It is also significant and positive for Swedish-born students with low socio-economic status (*p* < 0.001) but not with high socio-economic status (*p* = 0.607). It is significant and negative for all subgroups of immigrant students (girls: *p* < 0.001; boys: *p* = 0.002; high socioeconomic status: *p* = 0.003; low socioeconomic status: *p* < 0.001), indicating smaller relative increases in treatment for internalizing disorders among low-achieving immigrant students. Additional file C shows odds from the corresponding three-way interactions. The increase in the gap between low- and medium/high achieving students was, in relative terms, greater among Swedish-born compared to immigrant students (*p* < 0.001), and among Swedish-born students with low compared to with high socio-economic status (*p* = 0.012).

### Supplementary analyses

Logistic models with all subgroups combined showed that the association between low achievement and treatment for internalizing disorders in the pooled sample increased after adjusting for sex, country of birth and socio-economic status (Additional file E). Additive interactions were similar, but multiplicative interactions non-significant in all subgroups, for more severe forms of internalizing disorders (proxied by inpatient treatment; Additional file G). Analyses using a more fine-grained categorization of country of origin showed that the increase, in absolute as well as relative terms, in the association between achievement and treatment for internalizing disorders was generally greatest for Swedish-born students with Swedish-born parents. Analyses using a more fine-grained categorization of parental education showed that, in relative terms, the association between achievement and treatment for internalizing disorders changed in a non-linear way across parental education categories, while in absolute terms, the change was greatest for Swedish-born students of parents with at least three years post-secondary education and smallest for Swedish-born students of parents with less than upper secondary education (Additional file H).

## Discussion

The aim of this study was to investigate temporal changes between 2006 and 2018 in the association between academic achievements and treatment for internalizing disorders (henceforth *internalizing disorders* in short) among Swedish school year 9 students, and across subgroups defined by sex, country of birth, and socio-economic status. The main finding of the study was that temporal trends diverged strongly for Swedish-born and immigrant students. For Swedish-born students, the association between achievement and internalizing disorders became stronger over time in both absolute and relative terms, while among immigrant students, the association was largely stable in absolute terms and diminished in relative terms. Since the association was already stronger for Swedish-born students at the start of the period, this means that there was a growing divergence over time. Among Swedish-born students, the greatest increase in the association in absolute terms was observed in girls. Trends depending on socio-economic status were more complex, with the largest absolute increase in the association observed among Swedish-born students with high socio-economic status, but the largest relative increase observed among Swedish-born students with low socio-economic status.

A stronger association between low achievement and internalizing disorders, at least among Swedish-born students, is consistent with previous Swedish research. Jablonska et al. [18] examined students graduating from compulsory school in Stockholm between 2000 and 2007 and found that low achievement became a stronger risk factor for receiving disability benefits due to mental disorders as young adults. Högberg et al. [17] examined all compulsory school graduates between 1990 and 2018, and found that the association between achievement and inpatient treatment for internalizing disorders became stronger until around 2010, after which it continued to become stronger in absolute terms but not in relative terms. Högberg et al. did not adjust for or stratify by country of birth, and it is possible that the break in the increase after 2010 reflects the large increase in immigration to Sweden around that time. One consequence of this increase was that immigrants constituted a greater share of the low-achieving students, and the present study found that the association between achievement and internalizing disorders is smaller overall, and has not increased over time, among immigrant students. The findings of this study are only partly in line with a similar study by Nordmo et al. [36], using register data from Norway, which found a diminishing association between achievement and internalizing disorders in relative terms, though not in absolute terms, between 2006 and 2019. A key difference is that Nordmo et al. did not stratify by or adjust for country of birth. Indeed, results reported in Additional file E in the present study show that, in the pooled sample with all subgroups combined, the association between low achievement and internalizing disorders was largely stable over time in relative terms without any adjustment for covariates but became stronger over time when adjusting for demographic characteristics (including country of birth).

The present study was associational and all discussions regarding the causes of the observed associations are necessarily speculative. With this caveat in mind, one explanation for the stable or diminishing association among immigrant as compared to Swedish-born students may be differential selection into the low achievement-group. Immigration to Sweden increased strongly after 2006, and many of the immigrant children were from countries with low average educational attainment, arrived in Sweden at a relatively old age, and did not yet master the Swedish language by the end of compulsory school [37]. Both inadequate language skills and internalizing disorders are negatively associated with academic achievement [1, 2, 4, 37]. Thus, while low achievements among Swedish-born students may to a relatively greater extent be due to internalizing disorders, low achievements among immigrant students may increasingly have been a consequence of being new to the education system, thus generating a weaker association between achievement and internalizing disorders among immigrant students over time. Related to this, newly arrived immigrants may have lower “health literacy”, or a different understanding of mental health, and therefore a lower propensity to seek care for internalizing disorders [38]. Although speculative, this could be particularly pronounced among the most vulnerable segments of the immigrant population, with low academic achievements and low socio-economic status. Thus, the mostly stable or diminishing association between achievement and internalizing disorders among immigrant students may be a form of statistical artifact resulting from the changing composition of the immigrant group.

It is also possible that the stronger association between achievement and internalizing disorders observed among Swedish-born students is a form of statistical artifact, in the sense that temporal patterns in *observed* internalizing disorders do not reflect underlying symptoms but rather changing achievement-related differences in healthcare seeking behaviours or diagnostic practices. While we cannot rule out this possibility, we cannot, unlike in the case with immigrant students, think of any established societal trend that would substantiate it. If real and not a statistical artifact, the stronger association over time may reflect that internalizing disorders have become more detrimental for academic achievement, or that low achievements have become more distressing for students.

As for the first possibility (internalizing disorders being more detrimental for achievement), it is known that both academic achievement and internalizing disorders are influenced by genetic factors [39, 40], and that polygenic risk scores for achievement and internalizing disorders are correlated with other [41]. If, for whatever reason, the genetic correlates of achievement and internalizing disorders have become more clustered over time, we would expect low-achieving students to be an increasingly selected group in terms of innate vulnerability to internalizing disorders. This argument also corresponds with claims that growing health inequalities among working-age Europeans are partly due to stronger health-selection into low socio-economic status in more socially mobile societies [42]. It is also possible that internalizing disorders have become more strongly penalized in terms of achievement. For instance, students with internalizing disorders, and often comorbid externalizing problems, may find it more difficult to adapt to learning environments that, as in Sweden, increasingly emphasise independent work and students’ own responsibility for their learning [43].

As for the second possibility (low achievements being more distressing), growing economic inequality, deindustrialization, and rising returns on education [44] has increased the significance of academic achievements for, and exacerbated the detrimental impact of academic failure on, the social status and economic opportunities of adolescents [45]. Notably, economic inequality has risen more in Sweden than in almost any other high-income country in recent decades [46]. These changes, in turn, may have had a particularly strong impact on low-achieving students. Moreover, the Swedish education systems has simultaneously become more achievement-oriented due to educational reforms [47, 48] and the growth of large-scale international assessments, such as the Programme for International Student Assessment (PISA) [49]. The identities and sense of self-worth of low-achieving students may be more vulnerable to societal contexts where academic achievements are an important marker of social status [50, 51]. This explanation is also supported by the finding that the greatest increase was observed among Swedish-born girls. Girls tend to value school higher [19] and be more dependent on their achievements in school for their labour market outcomes [20]. They may therefore experience more distress from academic failures when achievement becomes more important for social status in the present as well as employment opportunities in the future.

### Limitations

We could only measure internalizing disorders using data on specialized psychiatric inpatient or outpatient care, or pharmacological treatment. We could not measure non-pharmacological treatment received in primary care, nor self-reported internalizing problems. Moreover, there may be underreporting of data from clinics to the National Patient Register [32]. Thus, in addition to underlying symptoms, our outcome measure also reflects differences in healthcare seeking behaviours, health literacy, diagnostic or treatment practices, or underreporting from clinics. Moreover, results based on clinically assessed diagnoses or pharmacological treatment may not be generalizable to symptoms of internalizing disorders that do not result in a diagnosis or treatment, nor to dimensional or self-reported measures of internalizing problems. Adolescents with internalizing disorders, or symptoms of such disorders, but who are not diagnosed or treated and thus not captured in the registers likely differ from adolescents that are diagnosed or treated. Moreover, these differences may have changed over the study period as the overall number of diagnosed or treated cases increased. We could not account for such unobserved differences in this study and cannot rule out that they may have influenced the results.

These limitations concerning the measurement of internalizing disorders are especially relevant considering the very large – almost four-fold – overall increase in these disorders during the studied period. Although self-reported internalizing problems among Swedish adolescents have also increased over the studied period [52], this increase has been more modest than the increase in treatment for internalizing disorders. Thus, the overall increase may partly be due to changing diagnostic or treatment practices, over and above a change in underlying symptoms. There are no known reasons to expect that diagnostic or treatment practices have changed more, or in different ways, for low-achieving students than for other adolescents, but we cannot rule out this possibility.

It is also possible that the measure of academic achievement, namely grades, influenced the results. Grades in Swedish schools are assigned locally by teachers and are not based on standardized tests, meaning that students with the same underlying ability may have different grades depending on which school they attend. For instance, students attending a relatively low-achieving school receive higher grades on average at a given level of underlying ability [53]. Thus, our measure of achievement may conflate underlying or absolute achievement with achievement relative to the school average. Relatedly, schools and their student composition can in themselves influence both achievement and mental health [54]. We did not have data on students’ schools and could not account for any such school effects.

The restricted temporal scope of the study – 13 years – is also a limitation. It is possible that the findings are unique to the period 2006–2018 and do not reflect a secular trend spanning decades. Lastly, the study was associational and does not allow for causal conclusions.

## Conclusions

This study investigated temporal trends in the association between academic achievement and internalizing disorders among Swedish students graduating from compulsory school (aged 16 years) between 2006 and 2018. It found an increasing association between achievement and internalizing disorders among Swedish-born students, most of all Swedish-born girls, but a stable or diminishing association among immigrant students. More research is needed to understand the causes of these diverging trends. For instance, studies utilizing quasi-experimental methods or a rich set of covariates may be able to disentangle whether trends are primarily due to internalizing disorders being more detrimental for academic achievement or if low achievement has become more distressing over time. Future research should also investigate whether these trends are limited to the Swedish context or if similar trends can be found in other countries, as well as whether the trends differ across different types of schools with different student compositions.

Abbreviations: GPA = grade point average; SES = socio-economic status. Internalizing disorder = diagnosed in specialized psychiatric inpatient or outpatient care and/or prescribed psychotropic drugs for internalizing problems.

## Supplementary Information

Supplementary Material 1.

## Data Availability

Data are not available for public use. Please contact Umeå SIMSAM Lab for further information on data availability.

## Abbreviations

ATC: Anatomical therapeutic chemical classification system
GPA: Grade point average
ICD: International classification of disease
SES: Socio-economic status
