# Supplementary material for: Associations between academic achievement and internalizing disorders in Sweden 2006–2018: Moderation by sex, socio-economic status, and country of birth
Source: BMC Pediatr. 2025 Oct 28;25:873. doi: 10.1186/s12887-025-06301-4 (PMC12560497; doi:10.1186/s12887-025-06301-4)
Supplement: Supplementary file 1 — Supplementary Material 1. [file 12887_2025_6301_MOESM1_ESM.docx]

**Additional files for *Associations between academic achievement and internalizing disorders in Sweden 2006-2018: Moderation by sex, socioeconomic status, and country of birth***

**Additional file A. Functional form of the association between achievement and internalizing disorders**

**Additional file B. Marginal effects corresponding to odds ratios in Table 2**

**Additional file D. Results for all GPA quintiles**

**Additional file C. Three-way interactions**

**Additional file E. Pooled sample with adjustment for moderators**

**Additional file F. GPA as outcome variable**

**Additional file G. Only inpatient care data**

**Additional file H. More fine-grained categorization of country of origin and parental education**

**Additional file A. Functional form of the association between achievement and internalizing disorders**

**Figure A1. Proportion with internalizing disorder by GPA percentile.**

Note. Abbreviations: GPA = grade point average. Estimates derived from logistic regression models.

**Additional file B. Marginal effects corresponding to odds ratios in Table 2**

**Table B1. Marginal effects of year on probability of internalizing disorders, by GPA. Stratified by country of birth**

|  | *Swedish-born* | *Immigrant* |
| --- | --- | --- |
| Medium/high GPA | 0.0034*** | 0.0025*** |
|  | [0.0034,0.0035] | [0.0022,0.0028] |
| Low GPA | 0.0132*** | 0.0032*** |
|  | [0.0129,0.0135] | [0.0028,0.0036] |
| Difference | 0.0097*** | 0.0007* |

Note. * p<0.05, ** p < 0.01, *** p<0.001. 95% confidence intervals in parentheses. Marginal effects derived from logistic regression models with internalizing disorder as the outcome. Difference = (Marginal effect for Low GPA) – (Marginal effect for Medium/high GPA). Abbreviations: GPA = grade point average.

**Table B2. Marginal effects of year on probability of internalizing disorders, by GPA. Stratified by country of birth and sex**

|  | *Swedish-born: Girls* | *Swedish-born: Boys* | *Immigrant: Girls* | *Immigrant: Boys* |
| --- | --- | --- | --- | --- |
| Medium/high GPA | 0.0046*** | 0.0023*** | 0.0030*** | 0.0020*** |
|  | [0.0044,0.0047] | [0.0022,0.0024] | [0.0025,0.0034] | [0.0017,0.0024] |
| Low GPA | 0.0183*** | 0.0103*** | 0.0030*** | 0.0034*** |
|  | [0.0178,0.0189] | [0.0100,0.0107] | [0.0023,0.0037] | [0.0028,0.0040] |
| Difference | 0.0137*** | 0.0080*** | 0.0000 | 0.0013*** |

Note. * p<0.05, ** p < 0.01, *** p<0.001. 95% confidence intervals in parentheses. Marginal effects derived from logistic regression models with internalizing disorder as the outcome. Difference = (Marginal effect for Low GPA) – (Marginal effect for Medium/high GPA). Abbreviations: GPA = grade point average.

**Table B3. Marginal effects of year on probability of internalizing disorders, by GPA. Stratified by country of birth and SES**

|  | *Swedish-born: High SES* | *Swedish-born: Low SES* | *Immigrant: High SES* | *Immigrant: Low SES* |
| --- | --- | --- | --- | --- |
| Medium/high GPA | 0.0033*** | 0.0032*** | 0.0023*** | 0.0020*** |
|  | [0.0032,0.0034] | [0.0030,0.0033] | [0.0019,0.0027] | [0.0016,0.0024] |
| Low GPA | 0.0141*** | 0.0115*** | 0.0025*** | 0.0017*** |
|  | [0.0134,0.0148] | [0.0111,0.0118] | [0.0016,0.0035] | [0.0012,0.0022] |
| Difference | 0.0108*** | 0.0083*** | 0.0002 | -0.0004 |

Note. * p<0.05, ** p < 0.01, *** p<0.001. 95% confidence intervals in parentheses. Marginal effects derived from logistic regression models with internalizing disorder as the outcome. Difference = (Marginal effect for Low GPA) – (Marginal effect for Medium/high GPA). Abbreviations: GPA = grade point average; SES = socio-economic status.

**Additional file C. Three-way interactions**

**Table C1. Odds of internalizing disorder by GPA, year and, respectively, country of birth, sex and SES.**

|  | *Country of birth* | *Swedish-born: Sex* | *Immigrant: Sex* | *Swedish-born: SES* | *Immigrant: SES* |
| --- | --- | --- | --- | --- | --- |
|  | OR [95% CI] | OR [95% CI] | OR [95% CI] | OR [95% CI] | OR [95% CI] |
| Year | 1.115*** | 1.148*** | 1.129*** | 1.145*** | 1.133*** |
|  | [1.103,1.128] | [1.142,1.154] | [1.107,1.151] | [1.139,1.151] | [1.108,1.159] |
| Low GPA | 2.600*** | 4.480*** | 3.465*** | 3.479*** | 3.283*** |
|  | [2.251,3.003] | [4.202,4.777] | [2.748,4.370] | [3.295,3.673] | [2.569,4.195] |
| Year X Low GPA | 0.965*** | 1.015*** | 0.963** | 1.016*** | 0.932*** |
|  | [0.951,0.980] | [1.007,1.022] | [0.940,0.986] | [1.009,1.023] | [0.906,0.958] |
|  |  |  |  |  |  |
| Swedish X Year | 1.020*** |  |  |  |  |
|  | [1.008,1.032] |  |  |  |  |
| Swedish X Low GPA | 1.481*** |  |  |  |  |
|  | [1.276,1.719] |  |  |  |  |
| Swedish X Year X Low GPA | 1.054*** |  |  |  |  |
|  | [1.037,1.071] |  |  |  |  |
|  |  |  |  |  |  |
| Girl X Year |  | 0.989*** | 0.982 |  |  |
|  |  | [0.983,0.995] | [0.959,1.006] |  |  |
| Girl X Low GPA |  | 0.944 | 0.672** |  |  |
|  |  | [0.871,1.023] | [0.498,0.906] |  |  |
| Girl X Year X Low GPA |  | 1.008 | 0.999 |  |  |
|  |  | [0.999,1.018] | [0.968,1.031] |  |  |
|  |  |  |  |  |  |
| High SES X Year |  |  |  | 0.992* | 0.971* |
|  |  |  |  | [0.986,0.999] | [0.944,0.999] |
| High SES X Low GPA |  |  |  | 1.601*** | 0.827 |
|  |  |  |  | [1.474,1.740] | [0.595,1.150] |
| High SES X Year X Low GPA |  |  |  | 0.987* | 1.031 |
|  |  |  |  | [0.976,0.997] | [0.992,1.071] |
| Constant | 0.010 | 0.006 | 0.006 | 0.011 | 0.006 |
| N | 1,422,411 | 1,279,919 | 142,492 | 1,186,793 | 116,817 |

Note. * p<0.05, ** p < 0.01, *** p<0.001. Abbreviations: OR = Odds ratio; CI = confidence interval; GPA = grade point average; SES = socio-economic status. Table presents results from logistic regression models.

**Table C2. Marginal effects of year on probability of internalizing disorders by GPA and, respectively, country of birth, sex and SES.**

|  | *Country of birth* | *Swedish-born: Sex* | *Immigrant: Sex* | *Swedish-born: SES* | *Immigrant: SES* |
| --- | --- | --- | --- | --- | --- |
| **Country of birth** |  |  |  |  |  |
| M-H GPA & Immigrant | 0.0025*** |  |  |  |  |
|  | [0.0022,0.0028] |  |  |  |  |
| M-H GPA & Swedish | 0.0034*** |  |  |  |  |
|  | [0.0034,0.0035] |  |  |  |  |
| Low GPA & Immigrant | 0.0032*** |  |  |  |  |
|  | [0.0028,0.0036] |  |  |  |  |
| Low GPA & Swedish | 0.0132*** |  |  |  |  |
|  | [0.0129,0.0135] |  |  |  |  |
| Second difference | 0.0090*** |  |  |  |  |
| **Sex** |  |  |  |  |  |
| M-H GPA & Boy |  | 0.0023*** | 0.0020*** |  |  |
|  |  | [0.0022,0.0024] | [0.0017,0.0024] |  |  |
| M-H GPA & Girl |  | 0.0046*** | 0.0030*** |  |  |
|  |  | [0.0044,0.0047] | [0.0025,0.0034] |  |  |
| Low GPA & Boy |  | 0.0103*** | 0.0034*** |  |  |
|  |  | [0.0100,0.0107] | [0.0028,0.0040] |  |  |
| Low GPA & Girl |  | 0.0183*** | 0.0030*** |  |  |
|  |  | [0.0178,0.0189] | [0.0023,0.0037] |  |  |
| Second difference |  | 0.0057*** | -0.0013* |  |  |
| **SES** |  |  |  |  |  |
| M-H GPA & Low SES |  |  |  | 0.0032*** | 0.0020*** |
|  |  |  |  | [0.0030,0.0033] | [0.0016,0.0024] |
| M-H GPA & High SES |  |  |  | 0.0033*** | 0.0023*** |
|  |  |  |  | [0.0032,0.0034] | [0.0019,0.0027] |
| Low GPA & Low SES |  |  |  | 0.0115*** | 0.0017*** |
|  |  |  |  | [0.0111,0.0118] | [0.0012,0.0022] |
| Low GPA & High SES |  |  |  | 0.0141*** | 0.0025*** |
|  |  |  |  | [0.0134,0.0148] | [0.0016,0.0035] |
| Second difference |  |  |  | 0.0024*** | 0.0005 |

Note. * p<0.05, ** p < 0.01, *** p<0.001. Abbreviations: OR = Odds ratio; CI = confidence interval; GPA = grade point average; M-H = medium/high.

Second differences are defined as:

- Country of birth: [(Low GPA & Swedish) – (M-H GPA & Swedish)] - [(Low GPA & Immigrant) – (M-H GPA & Immigrant)]
- Sex: [(Low GPA & Girl) – (M-H GPA & Girl)] - [(Low GPA & Boy) – (M-H GPA & Boy)]
- SES: [(Low GPA & High SES) – (M-H GPA & High SES)] - [(Low GPA & Low SES) – (M-H GPA & Low SES)]

**Additional file D. Results for all GPA quintiles**

Additional file C shows odds ratios from the logistic regression models that in turn were used to derive the marginal effects presented in Figure 2 in the main manuscript.

**Table D1. Odds of internalizing disorder by GPA and year. Stratified by country of birth.**

|  | *Swedish-born* | *Immigrant* |
| --- | --- | --- |
|  | OR [95% CI] | OR [95% CI] |
| Year | 1.157*** | 1.076*** |
|  | [1.154,1.161] | [1.066,1.087] |
| *GPA quintile* |  |  |
| 2nd | 0.317*** | 0.427*** |
|  | [0.301,0.335] | [0.347,0.525] |
| 3rd | 0.287*** | 0.400*** |
|  | [0.271,0.304] | [0.320,0.501] |
| 4th | 0.234*** | 0.356*** |
|  | [0.220,0.248] | [0.274,0.463] |
| 5th | 0.197*** | 0.336*** |
|  |  |  |
| *Year X GPA quintile* | [0.184,0.210] | [0.252,0.449] |
| Year X 2nd | 1.000 | 1.041*** |
|  | [0.994,1.006] | [1.019,1.063] |
| Year X 3rd | 0.979*** | 1.030* |
|  | [0.973,0.986] | [1.006,1.055] |
| Year X 4th | 0.975*** | 1.030* |
|  | [0.968,0.982] | [1.002,1.059] |
| Year X 5th | 0.974*** | 1.035* |
|  | [0.966,0.981] | [1.004,1.067] |
| Constant | 0.043 | 0.026 |
| N | 1,279,919 | 142,492 |

Note. * p<0.05, ** p < 0.01, *** p<0.001. Abbreviations: OR = Odds ratio; CI = confidence interval; GPA = grade point average. Table presents results from logistic regression models.

**Table D2. Odds of internalizing disorder by GPA and year. Stratified by country of birth and sex**

|  | *Swedish-born: Girls* | *Swedish-born: Boys* | *Immigrant: Girls* | *Immigrant: Boys* |
| --- | --- | --- | --- | --- |
|  | OR [95% CI] | OR [95% CI] | OR [95% CI] | OR [95% CI] |
| Year | 1.161*** | 1.165*** | 1.066*** | 1.087*** |
|  | [1.156,1.167] | [1.159,1.170] | [1.051,1.082] | [1.072,1.102] |
| *GPA quintile* |  |  |  |  |
| 2nd | 0.343*** | 0.274*** | 0.482*** | 0.368*** |
|  | [0.320,0.367] | [0.251,0.298] | [0.364,0.638] | [0.271,0.499] |
| 3rd | 0.284*** | 0.228*** | 0.488*** | 0.261*** |
|  | [0.265,0.305] | [0.206,0.252] | [0.369,0.646] | [0.176,0.386] |
| 4th | 0.205*** | 0.193*** | 0.393*** | 0.252*** |
|  | [0.191,0.221] | [0.171,0.217] | [0.287,0.539] | [0.154,0.411] |
| 5th | 0.157*** | 0.167*** | 0.349*** | 0.230*** |
|  | [0.145,0.170] | [0.144,0.194] | [0.250,0.487] | [0.121,0.436] |
| *Year X GPA quintile* |  |  |  |  |
| Year X 2nd | 0.996 | 1.003 | 1.045** | 1.039* |
|  | [0.989,1.004] | [0.994,1.013] | [1.015,1.076] | [1.008,1.072] |
| Year X 3rd | 0.975*** | 0.981*** | 1.028 | 1.042* |
|  | [0.967,0.983] | [0.970,0.992] | [0.998,1.059] | [1.001,1.085] |
| Year X 4th | 0.972*** | 0.972*** | 1.037* | 1.022 |
|  | [0.963,0.980] | [0.960,0.985] | [1.003,1.073] | [0.970,1.077] |
| Year X 5th | 0.973*** | 0.953*** | 1.046* | 1.004 |
|  | [0.964,0.981] | [0.937,0.969] | [1.010,1.083] | [0.937,1.076] |
| Constant | 0.067 | 0.028 | 0.032 | 0.022 |
| N | 624,194 | 655,725 | 67,292 | 75,200 |

Note. * p<0.05, ** p < 0.01, *** p<0.001. Abbreviations: OR = Odds ratio; CI = confidence interval; GPA = grade point average. Table presents results from logistic regression models.

**Table D3. Odds of internalizing disorder by GPA and year. Stratified by country of birth and SES**

|  | *Swedish-born: High SES* | *Swedish-born: Low SES* | *Immigrant: High SES* | *Immigrant: Low SES* |
| --- | --- | --- | --- | --- |
|  | OR [95% CI] | OR [95% CI] | OR [95% CI] | OR [95% CI] |
| Year | 1.139*** | 1.163*** | 1.057*** | 1.056*** |
|  | [1.131,1.146] | [1.158,1.168] | [1.036,1.078] | [1.038,1.074] |
| *GPA quintile* |  |  |  |  |
| 2nd | 0.244*** | 0.342*** | 0.404*** | 0.341*** |
|  | [0.223,0.267] | [0.319,0.368] | [0.294,0.555] | [0.244,0.477] |
| 3rd | 0.212*** | 0.295*** | 0.373*** | 0.402*** |
|  | [0.194,0.232] | [0.271,0.320] | [0.268,0.518] | [0.278,0.580] |
| 4th | 0.165*** | 0.247*** | 0.374*** | 0.180*** |
|  | [0.150,0.180] | [0.223,0.273] | [0.265,0.527] | [0.102,0.317] |
| 5th | 0.137*** | 0.213*** | 0.324*** | 0.204*** |
|  | [0.125,0.149] | [0.186,0.243] | [0.226,0.466] | [0.104,0.403] |
| *Year X GPA quintile* |  |  |  |  |
| Year X 2nd | 1.014* | 0.994 | 1.062** | 1.075*** |
|  | [1.003,1.026] | [0.986,1.003] | [1.023,1.102] | [1.035,1.117] |
| Year X 3rd | 0.996 | 0.979*** | 1.040* | 1.028 |
|  | [0.985,1.007] | [0.969,0.990] | [1.000,1.081] | [0.984,1.074] |
| Year X 4th | 0.992 | 0.973*** | 1.030 | 1.115*** |
|  | [0.981,1.003] | [0.960,0.986] | [0.988,1.073] | [1.047,1.188] |
| Year X 5th | 0.989* | 0.968*** | 1.028 | 1.123** |
|  | [0.978,1.000] | [0.951,0.985] | [0.985,1.073] | [1.040,1.211] |
| Constant | 0.064 | 0.037 | 0.034 | 0.022 |
| N | 605,082 | 581,711 | 53,969 | 62,848 |

Note. * p<0.05, ** p < 0.01, *** p<0.001. Abbreviations: OR = Odds ratio; CI = confidence interval; GPA = grade point average; SES = socio-economic status. Table presents results from logistic regression models.

**Additional file E. Pooled sample with adjustment for moderators**

Model 1: No adjustment.

Model 2: No adjustment, but restricted to observations with complete data on socio-demographic characteristics (meaning that the year 2018 is missing).

Model 3: Adjusted for sex, country of birth and socio-economic status, as well as all possible two- and three-way interactions of these.

Model 4: As model 1 + all two-way interactions between low GPA and, respectively, sex, country of birth and socio-economic status.

Model 5: As model 2 + all two-way interactions between year and, respectively, sex, country of birth and socio-economic status.

**Table E1. Odds of internalizing disorder by GPA and year.**

|  | *Model 1* | *Model 2* | *Model 3* | *Model 4* | *Model 5* |
| --- | --- | --- | --- | --- | --- |
|  | OR [95% CI] | OR [95% CI] | OR [95% CI] | OR [95% CI] | OR [95% CI] |
| Year X Low GPA | 0.995* | 0.996 | 1.009*** | 1.012*** | 1.009*** |
|  | [0.991,0.999] | [0.991,1.001] | [1.005,1.014] | [1.007,1.017] | [1.004,1.014] |
| Constant | 0.011 | 0.011 | 0.002 | 0.005 | 0.007 |
| N | 1,422,487 | 1,303,610 | 1,303,610 | 1,303,610 | 1,303,610 |

Note. * p<0.05, ** p < 0.01, *** p<0.001. Abbreviations: OR = Odds ratio; CI = confidence interval; GPA = grade point average. Table presents results from logistic regression models.

**Additional file F. GPA as outcome variable**

Additional file F shows results from multinomial logistic regression models, with GPA quintiles as the outcome variable and internalizing disorders as the focal independent variable.

**Table F1. Relative risks of GPA quintile. Stratified by country of birth.**

|  | *Swedish-born* | *Immigrant* |
| --- | --- | --- |
|  | RRR [95% CI] | RRR [95% CI] |
| *2nd quintile* |  |  |
| Year | 1.03*** | 0.99*** |
|  | [1.02,1.03] | [0.99,0.99] |
| Internalizing disorder | 0.31*** | 0.41*** |
|  | [0.29,0.32] | [0.33,0.51] |
| Year X Internalizing disorder | 1.01 | 1.05*** |
|  | [1.00,1.01] | [1.02,1.07] |
| *3rd quintile* |  |  |
| Year | 1.03*** | 0.97*** |
|  | [1.03,1.03] | [0.97,0.98] |
| Internalizing disorder | 0.27*** | 0.39*** |
|  | [0.26,0.29] | [0.31,0.49] |
| Year X Internalizing disorder | 0.99*** | 1.03** |
|  | [0.98,0.99] | [1.01,1.06] |
| *4th quintile* |  |  |
| Year | 1.03*** | 0.97*** |
|  | [1.03,1.03] | [0.96,0.97] |
| Internalizing disorder | 0.23*** | 0.36*** |
|  | [0.21,0.24] | [0.28,0.47] |
| Year X Internalizing disorder | 0.98*** | 1.03* |
|  | [0.97,0.98] | [1.00,1.06] |
| *5th quintile* |  |  |
| Year | 1.04*** | 0.97*** |
|  | [1.04,1.04] | [0.97,0.98] |
| Internalizing disorder | 0.19*** | 0.33*** |
|  | [0.18,0.20] | [0.25,0.45] |
| Year X Internalizing disorder | 0.98*** | 1.04* |
|  | [0.97,0.99] | [1.00,1.07] |
| N | 1,279,919 | 142,492 |

Note. * p<0.05, ** p < 0.01, *** p<0.001. Abbreviations: RRR = Relative risk ratio; CI = confidence interval; GPA = grade point average. Table presents results from multinomial logistic regression models. GPA quintile 1 is the reference category.

**Table F2. Relative risks of GPA quintile. Stratified by country of birth and sex.**

|  | *Swedish-born: Girls* | *Swedish-born: Boys* | *Immigrant: Girls* | *Immigrant: Boys* |
| --- | --- | --- | --- | --- |
|  | RRR [95% CI] | RRR [95% CI] | RRR [95% CI] | RRR [95% CI] |
| *2nd quintile* |  |  |  |  |
| Year | 1.03*** | 1.02*** | 1.00 | 0.98*** |
|  | [1.03,1.04] | [1.02,1.02] | [0.99,1.00] | [0.98,0.99] |
| Internalizing disorder | 0.34*** | 0.26*** | 0.46*** | 0.35*** |
|  | [0.31,0.36] | [0.24,0.28] | [0.35,0.62] | [0.26,0.48] |
| Year X Internalizing disorder | 1.00 | 1.01* | 1.05** | 1.04** |
|  | [0.99,1.01] | [1.00,1.02] | [1.02,1.08] | [1.01,1.08] |
| *3rd quintile* |  |  |  |  |
| Year | 1.04*** | 1.03*** | 0.99*** | 0.96*** |
|  | [1.04,1.04] | [1.02,1.03] | [0.98,0.99] | [0.96,0.97] |
| Internalizing disorder | 0.27*** | 0.21*** | 0.47*** | 0.26*** |
|  | [0.25,0.29] | [0.19,0.23] | [0.35,0.62] | [0.17,0.38] |
| Year X Internalizing disorder | 0.98*** | 0.99 | 1.03* | 1.04* |
|  | [0.97,0.99] | [0.98,1.00] | [1.00,1.07] | [1.00,1.09] |
| *4th quintile* |  |  |  |  |
| Year | 1.05*** | 1.03*** | 0.98*** | 0.96*** |
|  | [1.04,1.05] | [1.03,1.03] | [0.98,0.99] | [0.95,0.96] |
| Internalizing disorder | 0.20*** | 0.19*** | 0.39*** | 0.26*** |
|  | [0.19,0.22] | [0.17,0.21] | [0.28,0.53] | [0.16,0.43] |
| Year X Internalizing disorder | 0.97*** | 0.98*** | 1.04* | 1.02 |
|  | [0.97,0.98] | [0.96,0.99] | [1.00,1.08] | [0.96,1.07] |
| *5th quintile* |  |  |  |  |
| Year | 1.06*** | 1.03*** | 0.99** | 0.96*** |
|  | [1.05,1.06] | [1.03,1.03] | [0.99,1.00] | [0.95,0.96] |
| Internalizing disorder | 0.15*** | 0.16*** | 0.34*** | 0.24*** |
|  | [0.14,0.16] | [0.14,0.19] | [0.24,0.48] | [0.12,0.45] |
| Year X Internalizing disorder | 0.98*** | 0.96*** | 1.05** | 1.00 |
|  | [0.97,0.99] | [0.94,0.98] | [1.01,1.09] | [0.93,1.07] |
| N | 624,194 | 655,725 | 67,292 | 75,200 |

Note. * p<0.05, ** p < 0.01, *** p<0.001. Abbreviations: RRR = Relative risk ratio; CI = confidence interval; GPA = grade point average. Table presents results from multinomial logistic regression models. GPA quintile 1 is the reference category.

**Table F3. Relative risks of GPA quintile. Stratified by country of birth and SES.**

|  | *Swedish-born: High SES* | *Swedish-born: Low SES* | *Immigrant: High SES* | *Immigrant: Low SES* |
| --- | --- | --- | --- | --- |
|  | RRR [95% CI] | RRR [95% CI] | RRR [95% CI] | RRR [95% CI] |
| *2nd quintile* |  |  |  |  |
| Year | 1.03*** | 1.02*** | 1.00 | 0.99*** |
|  | [1.03,1.03] | [1.01,1.02] | [1.00,1.01] | [0.98,0.99] |
| Internalizing disorder | 0.24*** | 0.33*** | 0.40*** | 0.31*** |
|  | [0.22,0.26] | [0.31,0.35] | [0.29,0.55] | [0.22,0.45] |
| Year X Internalizing disorder | 1.02** | 1.00 | 1.06** | 1.09*** |
|  | [1.01,1.03] | [0.99,1.01] | [1.02,1.10] | [1.04,1.13] |
| *3rd quintile* |  |  |  |  |
| Year | 1.03*** | 1.01*** | 1.00 | 0.97*** |
|  | [1.03,1.03] | [1.01,1.01] | [0.99,1.01] | [0.96,0.97] |
| Internalizing disorder | 0.20*** | 0.28*** | 0.37*** | 0.39*** |
|  | [0.18,0.22] | [0.26,0.31] | [0.26,0.51] | [0.27,0.57] |
| Year X Internalizing disorder | 1.00 | 0.99* | 1.04* | 1.03 |
|  | [0.99,1.02] | [0.98,1.00] | [1.00,1.08] | [0.99,1.08] |
| *4th quintile* |  |  |  |  |
| Year | 1.03*** | 1.01*** | 1.00 | 0.96*** |
|  | [1.03,1.03] | [1.01,1.01] | [1.00,1.01] | [0.95,0.96] |
| Internalizing disorder | 0.16*** | 0.24*** | 0.38*** | 0.17*** |
|  | [0.15,0.17] | [0.22,0.27] | [0.27,0.53] | [0.10,0.31] |
| Year X Internalizing disorder | 1.00 | 0.98*** | 1.03 | 1.12*** |
|  | [0.99,1.01] | [0.96,0.99] | [0.99,1.07] | [1.05,1.20] |
| *5th quintile* |  |  |  |  |
| Year | 1.03*** | 1.01*** | 1.01*** | 0.94*** |
|  | [1.03,1.03] | [1.01,1.01] | [1.00,1.02] | [0.93,0.95] |
| Internalizing disorder | 0.13*** | 0.21*** | 0.32*** | 0.19*** |
|  | [0.12,0.14] | [0.18,0.24] | [0.23,0.46] | [0.10,0.39] |
| Year X Internalizing disorder | 1.00 | 0.97** | 1.03 | 1.13** |
|  | [0.98,1.01] | [0.95,0.99] | [0.99,1.07] | [1.04,1.23] |
| N | 605,082 | 581,711 | 53,969 | 62,848 |

Note. * p<0.05, ** p < 0.01, *** p<0.001. Abbreviations: RRR = Relative risk ratio; CI = confidence interval; GPA = grade point average; SES = socio-economic status. Table presents results from multinomial logistic regression models. GPA quintile 1 is the reference category.

**Additional file G. Only inpatient care data**

**Table G1. Odds of internalizing disorder by GPA and year.**

|  | *Swedish-born* | *Immigrant* | *Swedish-born: Girls* | *Swedish-born: Boys* | *Swedish-born: High SES* | *Swedish-born: Low SES* |
| --- | --- | --- | --- | --- | --- | --- |
|  | OR [95% CI] | OR [95% CI] | OR [95% CI] | OR [95% CI] | OR [95% CI] | OR [95% CI] |
| Year | 1.067*** | 1.060** | 1.067*** | 1.071*** | 1.071*** | 1.070*** |
|  | [1.053,1.081] | [1.015,1.108] | [1.051,1.083] | [1.041,1.102] | [1.050,1.093] | [1.046,1.094] |
| Low GPA | 5.124*** | 2.505*** | 6.553*** | 4.628*** | 7.728*** | 4.350*** |
|  | [4.425,5.934] | [1.498,4.191] | [5.548,7.740] | [3.391,6.317] | [6.119,9.759] | [3.529,5.362] |
| Year X Low GPA | 0.991 | 0.992 | 0.996 | 0.993 | 0.976 | 0.992 |
|  | [0.973,1.009] | [0.938,1.050] | [0.975,1.016] | [0.956,1.032] | [0.946,1.007] | [0.963,1.021] |
| Constant | 0.001 | 0.001 | 0.001 | 0.000 | 0.001 | 0.001 |
| N | 1,279,919 | 142,492 | 624,194 | 655,725 | 605,082 | 581,711 |

Note. * p<0.05, ** p < 0.01, *** p<0.001. Abbreviations: OR = Odds ratio; CI = confidence interval; GPA = grade point average; SES = socio-economic status. Table presents results from logistic regression models.

**Table G2. Marginal effects of year on probability of internalizing disorders, by GPA.**

|  | *Swedish-born* | *Immigrant* | *Swedish-born: Girls* | *Swedish-born: Boys* | *Swedish-born: High SES* | *Swedish-born: Low SES* |
| --- | --- | --- | --- | --- | --- | --- |
| Medium/High GPA | 0.0001*** | 0.0001* | 0.0001*** | 0.0000*** | 0.0001*** | 0.0001*** |
|  | [0.0001,0.0001] | [0.0000,0.0001] | [0.0001,0.0001] | [0.0000,0.0001] | [0.0001,0.0001] | [0.0001,0.0001] |
| Low GPA | 0.0003*** | 0.0002** | 0.0007*** | 0.0001*** | 0.0003*** | 0.0003*** |
|  | [0.0002,0.0004] | [0.0000,0.0003] | [0.0005,0.0008] | [0.0001,0.0002] | [0.0002,0.0005] | [0.0002,0.0004] |
| Difference | 0.0002*** | 0.0001 | 0.0006*** | 0.0001** | 0.0003** | 0.0002*** |

Note. * p<0.05, ** p < 0.01, *** p<0.001. 95% confidence intervals in parentheses. Marginal effects derived from logistic regression models with internalizing disorder as the outcome. Difference = (Marginal effect for Low GPA) – (Marginal effect for Medium/high GPA). Abbreviations: GPA = grade point average; SES = socio-economic status.

**Additional file H. More fine-grained categorization of country of origin and parental education**

Country of origin is categorized into:

- Swedish = Swedish-born students with two Swedish-born parents
- Swedish-born, Western = Swedish-born students with a Western background (at least one parent born in, but no parent born outside, Europe or North America)
- Swedish-born, non-Western = Swedish-born students with a non-Western background (at least one parent born outside Europe or North America)
- Foreign-born, Western = immigrant students with a Western background (at least one parent born in, but no parent born outside, Europe or North America)
- Foreign-born, non-Western = immigrant students with a non-Western background (at least one parent born outside Europe or North America)

**Table H1. Odds of internalizing disorder by GPA, year and country of origin.**

|  | *OR* | *95% CI* |
| --- | --- | --- |
| Year | 1.142*** | [1.139,1.146] |
| Low GPA | 3.985*** | [3.820,4.156] |
| Year X Low GPA | 1.021*** | [1.016,1.026] |
| *Country background (ref: Swedish)* |  |  |
| Swedish-born, Western | 1.128** | [1.040,1.223] |
| Swedish-born, non-Western | 0.804*** | [0.719,0.900] |
| Foreign-born, Western | 1.067 | [0.931,1.223] |
| Foreign-born, non-Western | 0.647*** | [0.531,0.788] |
| *Year X Country background* |  |  |
| Year X Swedish-born, Western | 0.982*** | [0.973,0.991] |
| Year X Swedish-born, non-Western | 0.991 | [0.979,1.002] |
| Year X Foreign-born, Western | 0.991 | [0.977,1.006] |
| Year X Foreign-born, non-Western | 0.964*** | [0.945,0.984] |
| *Low GPA X Country background* |  |  |
| Low GPA X Swedish-born, Western | 0.816*** | [0.726,0.917] |
| Low GPA X Swedish-born, non-Western | 0.859 | [0.732,1.008] |
| Low GPA X Foreign-born, Western | 0.717*** | [0.591,0.870] |
| Low GPA X Foreign-born, non-Western | 0.618*** | [0.482,0.793] |
| *Year X Low GPA X Country background* |  |  |
| Year X Low GPA X Swedish-born, Western | 0.996 | [0.983,1.010] |
| Year X Low GPA X Swedish-born, non-Western | 0.981* | [0.964,0.998] |
| Year X Low GPA X Foreign-born, Western | 0.950*** | [0.930,0.971] |
| Year X Low GPA X Foreign-born, non-Western | 0.950*** | [0.926,0.974] |
| Constant | 0.011 |  |
| N | 1,416,761 |  |

Note. * p<0.05, ** p < 0.01, *** p<0.001. Abbreviations: OR = Odds ratio; CI = confidence interval; GPA = grade point average. Table presents results from logistic regression models.

**Table H2. Marginal effects of year on probability of internalizing disorders, by GPA and country of origin.**

|  | *B* | *95% CI* |
| --- | --- | --- |
| M-H GPA & Swedish | 0.0036*** | [0.0035,0.0037] |
| M-H GPA & Swedish-born, Western | 0.0031*** | [0.0028,0.0033] |
| M-H GPA & Swedish-born, non-Western | 0.0028*** | [0.0026,0.0031] |
| M-H GPA & Foreign-born, Western | 0.0036*** | [0.0031,0.0040] |
| M-H GPA & Foreign-born, non-Western | 0.0015*** | [0.0012,0.0019] |
| Low GPA & Swedish | 0.0146*** | [0.0142,0.0150] |
| Low GPA & Swedish-born, Western | 0.0105*** | [0.0097,0.0112] |
| Low GPA & Swedish-born, non-Western | 0.0084*** | [0.0075,0.0092] |
| Low GPA & Foreign-born, Western | 0.0054*** | [0.0045,0.0064] |
| Low GPA & Foreign-born, non-Western | 0.0019*** | [0.0015,0.0024] |
| *Second difference vs. Swedish* |  |  |
| Swedish-born, Western | -0.0036*** |  |
| Swedish-born, non-Western | -0.0055*** |  |
| Foreign-born, Western | -0.0091*** |  |
| Foreign-born, non-Western | -0.0106*** |  |

Note. * p<0.05, ** p < 0.01, *** p<0.001. Abbreviations: OR = Odds ratio; CI = confidence interval; GPA = grade point average; M-H = medium/high.

Second differences are defined as:

- [(Low GPA & Swedish) – (M-H GPA & Swedish)] - [(Low GPA & Swedish-born, Western) – (M-H GPA & Swedish-born, Western)]
- [(Low GPA & Swedish) – (M-H GPA & Swedish)] - [(Low GPA & Swedish-born, non-Western) – (M-H GPA & Swedish-born, non-Western)]
- [(Low GPA & Swedish) – (M-H GPA & Swedish)] - [(Low GPA & Foreign-born, Western) – (M-H GPA & Foreign-born, Western)]
- [(Low GPA & Swedish) – (M-H GPA & Swedish)] - [(Low GPA & Foreign-born, non-Western) – (M-H GPA & Foreign-born, non-Western)]

**Table H3. Odds of internalizing disorder by GPA, year and parental education.**

|  | *OR* | *95% CI* |
| --- | --- | --- |
| Year | 1.151*** | [1.126,1.177] |
| Low GPA | 3.153*** | [2.568,3.870] |
| Year X Low GPA | 1.000 | [0.974,1.027] |
| *Parental education (ref: Lower secondary)* |  |  |
| Upper secondary | 1.066 | [0.894,1.271] |
| Post secondary < 3 years | 1.039 | [0.867,1.246] |
| Post secondary ≥ 3 years | 1.235* | [1.035,1.474] |
| *Year X Parental education* |  |  |
| Year X Upper secondary | 0.995 | [0.972,1.018] |
| Year X Post secondary < 3 years | 0.992 | [0.970,1.016] |
| Year X Post secondary ≥ 3 years | 0.983 | [0.961,1.006] |
| *Low GPA X Country background* |  |  |
| Low GPA X Upper secondary | 1.128 | [0.912,1.395] |
| Low GPA X Post secondary < 3 years | 1.709*** | [1.362,2.145] |
| Low GPA X Post secondary ≥ 3 years | 1.882*** | [1.509,2.347] |
| *Year X Low GPA X Country background* |  |  |
| Year X Low GPA X Upper secondary | 1.017 | [0.989,1.045] |
| Year X Low GPA X Post secondary < 3 years | 0.998 | [0.969,1.028] |
| Year X Low GPA X Post secondary ≥ 3 years | 1.004 | [0.976,1.033] |
| Constant | 0.010 |  |
| N | 1,175,175 |  |

Note. * p<0.05, ** p < 0.01, *** p<0.001. Abbreviations: OR = Odds ratio; CI = confidence interval; GPA = grade point average. Table presents results from logistic regression models.

**Table H4. Marginal effects of year on probability of internalizing disorders, by GPA and parental education.**

|  | *B* | *95% CI* |
| --- | --- | --- |
| M-H GPA X Lower secondary | 0.0030*** | [0.0025,0.0036] |
| M-H GPA X Upper secondary | 0.0032*** | [0.0030,0.0033] |
| M-H GPA X Post secondary < 3 years | 0.0031*** | [0.0029,0.0033] |
| M-H GPA X Post secondary ≥ 3 years | 0.0034*** | [0.0032,0.0035] |
| Low GPA X Lower secondary | 0.0086*** | [0.0077,0.0096] |
| Low GPA X Upper secondary | 0.0118*** | [0.0114,0.0122] |
| Low GPA X Post secondary < 3 years | 0.0128*** | [0.0118,0.0138] |
| Low GPA X Post secondary ≥ 3 years | 0.0152*** | [0.0142,0.0163] |
| *Second difference vs. Lower secondary* |  |  |
| Upper secondary | 0.0031*** |  |
| Post secondary < 3 years | 0.0041*** |  |
| Post secondary ≥ 3 years | 0.0062*** |  |

Note. * p<0.05, ** p < 0.01, *** p<0.001. Abbreviations: OR = Odds ratio; CI = confidence interval; GPA = grade point average; M-H = medium/high.

Second differences are defined as:

- [(Low GPA & Lower secondary) – (M-H GPA & Lower secondary)] - [(Low GPA & Upper secondary) – (M-H GPA & Upper secondary)]
- [(Low GPA & Lower secondary) – (M-H GPA & Lower secondary)] - [(Low GPA & Post secondary < 3 years) – (M-H GPA & Post secondary < 3 years)]
- [(Low GPA & Lower secondary) – (M-H GPA & Lower secondary)] - [(Low GPA & Post secondary ≥ 3 years) – (M-H GPA & Post secondary ≥ 3 years)]
